# Integrated Conductive Hybrid Electrode Materials Based on PPy@ZIF-67-Derived Oxyhydroxide@CFs Composites for Energy Storage

**DOI:** 10.3390/polym13071082

**Published:** 2021-03-29

**Authors:** Shuaishuai Yang, Xianhui An, Xueren Qian

**Affiliations:** Key Laboratory of Bio-Based Material Science & Technology, Northeast Forestry University, Ministry of Education, Harbin 150040, China; yangshuaishuai10@163.com (S.Y.); anxianh509@163.com (X.A.)

**Keywords:** cellulose fibers, ZIF-67 derived cobalt oxyhydroxide, polypyrrole, energy storage

## Abstract

Due to excellent flexibility and hydrophilicity, cellulose fibers (CFs) have become one of the most potential substrate materials in flexible and wearable electronics. In previous work, we prepared cobalt oxyhydroxide with crystal defects modified polypyrrole (PPy)@CFs composites with good electrochemical performance. In this work, we redesigned the crystalline and nanoscale cobalt oxyhydroxide with zeolitic imidazolate frameworks-67 (ZIF-67) as precursor. The results showed that the PPy@ZIF-67 derived cobalt oxyhydroxide@CFs (PZCC) hybrid electrode materials possess far better capacitance of 696.65 F·g^−1^ than those of PPy@CFs (308.75 F·g^−1^) and previous PPy@cobalt oxyhydroxide@CFs (571.3 F·g^−1^) at a current density of 0.2 A·g^−1^. The PZCC delivers an excellent cyclic stability (capacitance retention of 92.56%). Moreover, the PZCC-supercapacitors (SCs) can provide an energy density of 45.51 mWh cm^−3^ at a power density of 174.67 mWh·cm^−3^, suggesting the potential application in energy storage area.

## 1. Introduction

With the rapid development of portable and wearable products, the demand for the flexible energy storage devices will also increase to meet the growth of flexible electronics. Among them, supercapacitors (SCs) with high power density, long cycling life, and excellent charge/discharging ability, have attracted researchers’ interests [1,2,3,4,5,6,7,8,9]. Based on different mechanisms of energy storage, the SCs electrode materials can be divided into electrical double-layer capacitor materials (EDLCs) such as activated carbon [10], carbon nanotubes [11], graphene [12,13], etc. and pseudo-capacitor materials (PCs), such as metal oxides [14,15], metal sulfides [16], conductive polymers [17,18], etc. It should be noted that electrode material is one of the most important components for SCs, which directly determines the electrochemical performances. In practical application, flexible electrode materials should not only meet high efficiency energy storage, but also satisfy low costs and environmental friendliness. Therefore, the development of new high-performance flexible electrode materials has a very important significance for electronic products.

Cellulose fibers (CFs) as superior flexible substrate materials contain rich hydroxyl functional groups and abundant raw material sources, are environmentally friendly, sustainable, light-weight, high-strength, low-cost, recyclable and have good flexibility. Therefore, we select them as substrate materials [19,20,21]. The porous and rough surface structure provides excellent substrate materials for the loading of electrode materials and the construction of flexible SCs. At the same time, the good wettability of cellulose fiber provides powerful conditions for the electrochemical transfer process between the electrode material and the electrolyte [22]. These unique properties lay the foundation for the construction of flexible SCs.

Compared to the EDLCs, the PCs have higher specific capacitance and energy density due to their reversible redox process. In numerous PCs, conductive polymers (CPs) such as polyaniline (PANI) [23], polypyrrole (PPy) [24], polyinode (PIn) [25], and Poly(3,4-ethylenedioxythiophene) (PEDOT) [26], have been favored by researchers because of their good electrochemical performance, facile synthesis, and excellent environmental stability. Among them, the PPy with good conductivity and specific capacitance has been widely studied, which mainly focused on the modification of its micromorphology via template method, and combination with other materials, such as PPy/carbon nanotubes (CNTs) [27,28], PPy/graphene oxide (GO) [29,30], PPy/Ti_3_C_2_T_x_ [31,32], PPy/MnO_2_ [33,34], PPy/CuS [35], etc. In this work, we proposed to prepare high performance PPy@CFs electrode from the perspective of interface modification between cellulose fibers (CFs) and PPy.

In previous studies, our group investigated the effect of cobalt oxyhydroxide via the NaBH_4_ reduction method on the electrochemical performance of PPy@CFs composite electrode [36]. The results indicated that the composite electrode exhibited good specific capacitance and cyclic stability. However, we obtained cobalt oxyhydroxide via the method with irregular and amorphous particles. Herein, we conceive to design a cobalt compound with a certain structure as precursor to prepare cobalt oxyhydroxide with high crystallinity of nanometer-scale, which could be more preferred for the electrochemical process of PPy@CFs composites. It is well known that metal-organic frameworks (MOFs) derived materials have aroused widespread concern due to their characteristics, which are organic-inorganic hybrid materials with intramolecular pores formed by self-assembly of organic ligands and metal ions or clusters through coordination bonds. They have many advantages, for example, they are porous, large, and have a specific surface area, making them more conducive to their application of SCs. Therefore, in this paper, we chose ZIF-67 as precursor to prepare cobalt oxyhydroxide with high crystalline and nanoscale in the presence of CFs via the in situ oxidation method, and then coated the PPy on the surface of composites to optimize the electrochemical performance.

## 2. Experiment

### 2.1. Materials and Reagents

Cobalt nitrate, 2-methylimidazole, ammonium persulfate, and ferric chloride were of analytical grade and used as received. Pyrrole monomer was purified by distillation before use. Canada market bleached softwood kraft pulp as CFs source was provided by Mudanjiang Hengfeng Paper Co. Ltd. (Heilongjiang, China) and beaten to 37 °SR before use.

### 2.2. Preparation of ZIF-67 Derived Cobalt Oxyhydroxide@CFs Composites

The ZIF-67 derived cobalt oxyhydroxide@CFs composites were prepared by a simple in-situ method. Briefly, 0.5 g of CFs were dispersed in a water or water/methanol system to obtain CFs suspension. Then, the cobalt nitrate (1 mmol) and 2-methylimidazole were added according to the molar ratio of 1:10, and the reaction was carried out at room temperature for 12 h to obtain ZIF-67@CFs composites. Finally, the ZIF-67 derived cobalt oxyhydroxide@CFs composites were prepared via the oxidation process of ammonium persulfate at 95 °C for 10 h. The ZIF-67 derived cobalt oxyhydroxide@CFs in water phase was marked as ZCC-1, and the ZIF-67 derived cobalt oxyhydroxide@CFs in water/methanol (volume ratio of 1:1) system was marked as ZCC-2.

### 2.3. Preparation of PPy@ZIF-67 Derived Cobalt Oxyhydroxide@CFs Composites

The typical preparation process was as follows: 0.25 mL of pyrrole monomer was dispersed in the ZCC-1 and ZCC-2 suspension, and then 0.973 g of ferric chloride as initiator was added into the suspension under continuous stirring for 6 h in an ice-bath system. After the reaction, the mixture was filtrated with distilled water until it was colorless. The PPy@ZIF-67 derived cobalt oxyhydroxide@CFs composites were prepared and noted as PZCC-1 and PZCC-2.

### 2.4. Characterizations

The morphology of electrode materials was observed by a scanning electron microscope (SEM, SEU 8010). The structure and chemical valence were investigated through X-ray diffraction (XRD, X’ Pert3 Powder) at a range of 5° to 80° with a scan rate of 5° min^−1^, fourier transform infrared spectroscopy (FT-IR, Bruker Vertex 80V) with a scanning range of 4000–400 cm^−1^ at a resolution of 4 cm^−1^, and X-ray photoelectron spectroscopy (XPS, Thermo ESCALAB 250XI), respectively. Thermal gravimetric analysis (TGA) was performed by STA449 F3 equipment from room temperature to 800 °C in air with a heating rate of 10 °C/min^−1^. The contact angle was measured by JY-82B Kruss DSA equipment.

### 2.5. Electrochemical Measurements

The measurements of cyclic voltammetry (CV), galvanostatic charge-discharge (GCD), and electrochemical impedance spectroscopy (EIS) were carried out in a three-electrode system in 0.6 M H_2_SO_4_ electrolyte. The prepared composite electrodes, Pt electrode, and Ag/AgCl in 1 M KCl were used as working electrode, counter electrode, and reference electrode, respectively. The specific capacitance of composite electrode was calculated from GCD profiles via the equations:

(1) $$C_{s}=\frac{\int IdV}{2m\nu \Delta V}$$

(2) $$C_{m}=\frac{I\Delta t}{m\Delta V}$$

where *C*_s_ and *C*_m_ are specific capacitance (F·g^−1^) for CV and GCD profiles, and *m*, *ν*, *I*, Δ*V,* and Δ*t* represent active mass (g), scan rate (V·s^−1^), current density (A·g^−1^), potential window, and discharge time (s), respectively.

In a two-electrode system, the two same electrodes were assembled into the SCs. The energy density and power density were calculated according to the following equations [37]:

(3) $$C_{a}=\frac{I\Delta t}{A\Delta V}$$

(4) $$E=\frac{C_{a}\times \Delta V^{2}}{2\times 3600\times d}$$

(5) $$P=\frac{3600E}{\Delta t}$$

where *C*_a_, *I*, *A*, Δ*t,* and Δ*V* represent specific capacitance (mF·cm^−2^), current density (mA cm^−2^), active area (cm^2^) of two electrodes, discharge time (s), and potential window, respectively, and *E* (mWh·cm^−3^) and *P* (mW·cm^−3^) refer to energy density and power density, respectively.

## 3. Results and Discussion

### 3.1. Fabrication Process

**Figure 1:**
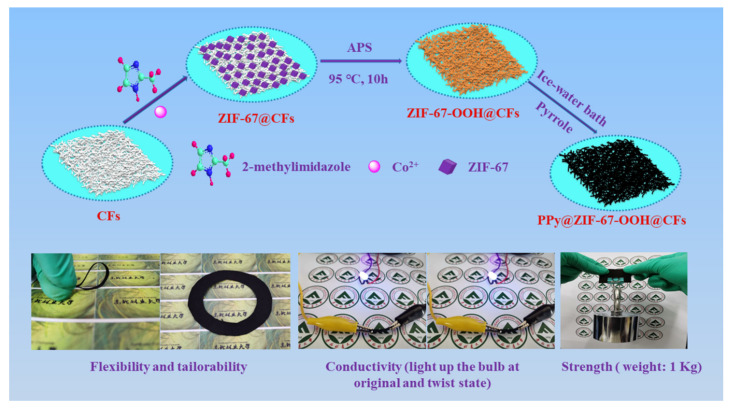
Fabrication process of PPy@ZIF-67 derived cobalt oxyhydroxide@CFs (PZCC) hybrid electrode materials and photographic images (flexibility, tailorability, conductivity, and strength).

The fabrication process of PZCC hybrid electrode materials and photographic images (flexibility, tailorability, conductivity, and strength) are shown in Figure 1. Cellulose with polar hydroxyl and glycoaldehyde acid group makes the surface show electronegativity, which will facilitate the adsorption of Co^2+^ on the surface of cellulose fibers via electrostatic force. The preparation process for hybrid electrode materials are as follows. Firstly, the CFs suspension, cobalt nitrate, and 2-methylimidazole were mixed in water or water/methanol under a continuous stirring system for 12 h at room temperature. Secondly, the ZCC was obtained by adding APS as oxidant at 95 °C for 10 h. Then, a facile in-situ oxidative polymerization method was performed to prepare PZCC hybrid electrode materials under an ice-water bath condition. The chemical bonding force between cobalt oxyhydroxide and PPy can make PPy anchor firmly on cobalt oxyhydroxide@CFs composite. Due to the excellent flexibility, tailorability, conductivity, and strength of PZCC hybrid electrode materials, it can be applied in flexible energy storage area.

### 3.2. Characterization Analysis

**Figure 2:**
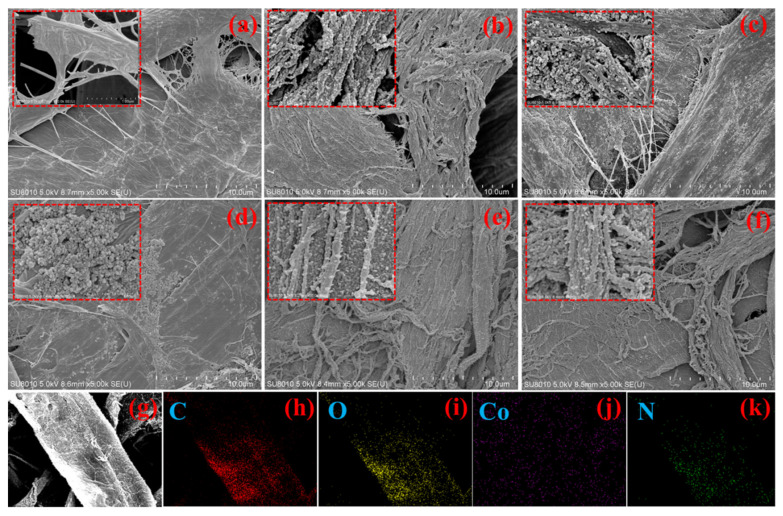
SEM images of cellulose fibers (CFs) (**a**), pseudo-capacitor materials (PC) (**b**), ZCC-1 (**c**), ZCC-2(**d**), PZCC-1(**e**), and PZCC-2 (**f**). Elemental mapping of PZCC-2 (**g**–**k**).

The morphology of CFs, PC, ZCC-1, ZCC-2, PZCC-1, and PZCC-2 at low and high magnifications (insert images) are shown in Figure 2. The surface of pure CFs is smooth, with no other substance in Figure 2a. The morphology of PC composite is displayed in Figure 2b, where the PPy presents a spherical shape with a diameter of tens of nanometers. Figure 2c,d shows the rough surface of CFs at low magnification, indicating the successful coating of cobalt oxyhydroxide, and it can be seen clearly that some particles are tightly packed and distributed on the surface of CFs at high magnification. The SEM results showed that the successful preparation of cobalt oxyhydroxide particles with regular nanoscale via ZIF-67 as precursor. Compared to PPy@CFs composites, the microscopic morphology of PZCC hybrid electrode material is different, as shown in Figure 2e,f, which is mainly reflected in the change from spherical to short rod morphology. This is more conducive to the charge transfer in the electrochemical process. The elemental mapping of PZCC-2 hybrid electrode material provides more evidence for homogeneous distribution of Co and N elements, i.e., cobalt oxyhydroxide and PPy, as shown in Figure 2g–k.

**Figure 3:**
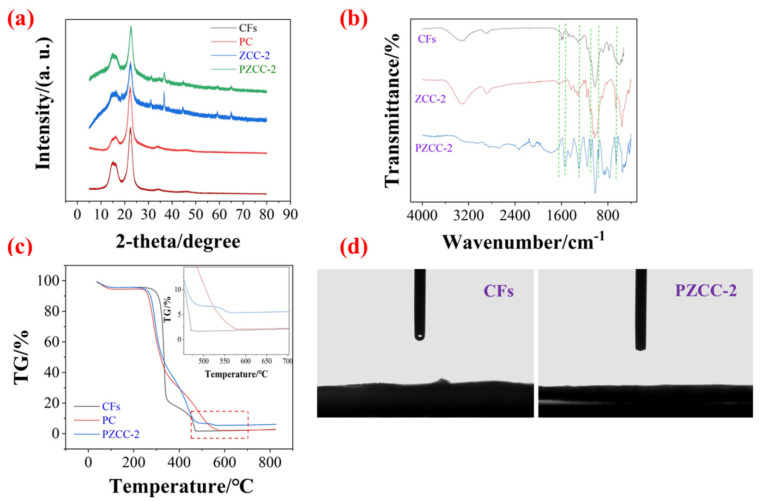
(**a**) XRD patterns of CFs, PC, ZCC-2, and PZCC-2. (**b**) FTIR spectra of CFs, ZCC-2, and PZCC-2. (**c**) TG curves of CFs, PC, and PZCC-2. (**d**) Hydrophilicity of CFs and PZCC-2.

To analyze the crystallographic structure of composites, the X-ray diffraction (XRD) test was performed as shown in Figure 3a. The diffraction peaks at 15.6° and 22.5° are attributable to the characteristic peaks of CFs. In addition, the characteristic peak of PPy in PC composites is about 22.9° [38], which coincided with the (002) diffraction plane of CFs. There were some extra peaks in ZCC-2 and PZCC-2 composites at 18.8°, 39.1°, 44.3°, and 65.1°, corresponding to the (003), (111), (140), and (002) planes of cobalt oxyhydroxide (ICDD/JCPDS 26-0480, space group: Pbnm (62), a = 4.353 nm, b = 9.402 nm, c = 2.840 nm). Compared to the previous NaBH_4_ reduction method, this strategy obtained cobalt oxyhydroxide with higher crystallinity, which may be more helpful for the electrochemical process. The FTIR spectroscopy can be used to confirm the formation of CFs, ZCC-2, and PZCC-2 composites, and reveal the intermolecular binding force (Figure 3b). The peaks at 3350, 2901, and 1028 cm^−1^ are attributed to O–H, C–H, and C–O–C, respectively [35]. The additional peaks at 1637 and 657 cm^−1^ in the ZCC-2 composite are characteristic peaks of cobalt oxyhydroxide, attributing to Co–O and Co–O^2−^, respectively [39,40]. As for PZCC-2, those at 1543, 1295, 1095, and 964 cm^−1^ can be assigned to C=C, C–N, N–H, and C–C of the PPy ring, respectively [41,42,43,44]. The thermal stability and hyrdrophilicity of the composites were characterized by a thermal analyzer and contact angle tester, shown in Figure 3c,d. The results suggest that PZCC-2 hybrid electrode material possesses a better thermal stability, demonstrating that the loading of cobalt oxyhydroxide and PPy is beneficial to its thermal stability. It is well known that the hydrophilicity of electrode material is a crucial parameter in the electrochemical process, only when the electrode material has good hydrophilicity, which allows for better contact with the electrolyte. Based on this opinion, CFs can be well used in the preparation of flexible electrode materials. Figure 3d shows that PZCC-2 has far better hydrophilicity than that of CFs, indicating its potential application in the flexible electronics field.

**Figure 4:**
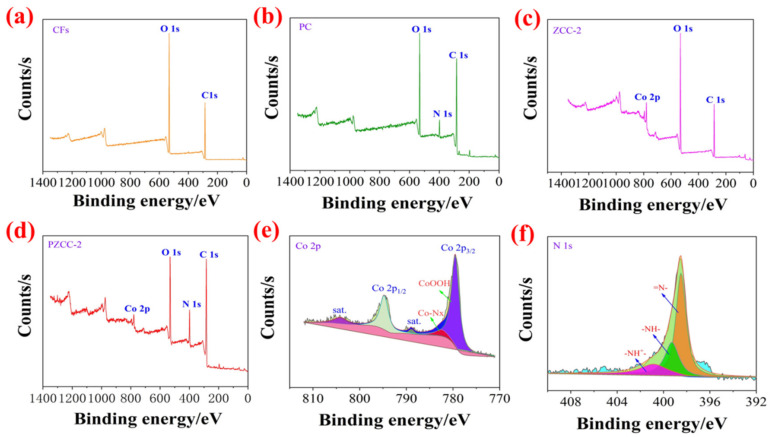
XPS survey spectrum of CFs (**a**), PC (**b**), ZCC-2 (**c**), and PZCC-2 (**d**). High-resolution XPS of Co 2p (**e**) and N 1s (**f**) based on ZCC-2 and PZCC-2.

The X-ray photoelectron spectroscopy (XPS) was carried out in order to further investigate the elemental composition and chemical properties of composites. As shown in Figure 4, compared to different composites, the C, N, O, Co, elements in PZCC-2 hybrid electrode material originate from CFs (C and O elements), PPy (N element), and cobalt oxyhydroxide (Co element), which indicates that cobalt oxyhydroxide and PPy were successfully loaded on the surface of CFs. As presented in Figure 4e, the Co 2p spectrum is deconvoluted into two main peaks with satellite peak of Co 2p1/2 and Co 2p3/2 at 794.2 and 779.2 eV. The XPS Co2p3/2 high-resolution spectrum of PZCC-2 can be fitted into three peaks at 779.6 eV, 782.4 eV, and 789.0 eV, attributing to Co–O, Co–Nx, and shake-up satellite peaks, which demonstrates the existence of cobalt oxyhydroxide and Co-N coordination bond simultaneously [45,46]. In addition, from Figure 4f, the XPS N 1s spectrum of high-resolution spectrum of PZCC-2 can be fitted into three peaks at 398.5 eV for =N–, 399.3 eV for –NH–, and 401.0 eV for -NH^+^, suggesting the successful loading of PPy in PZCC-2 hybrid electrode material.

### 3.3. Evaluation of Electrochemical Performance

**Figure 5:**
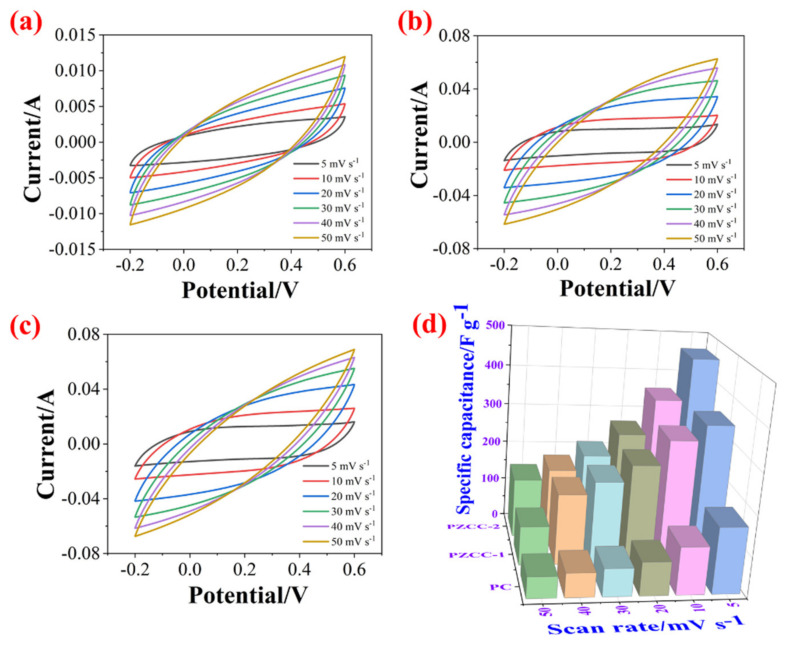
CV profiles of PC (**a**), PZCC-1 (**b**), and PZCC-2 (**c**) electrodes recorded using different scan rates of 5, 10, 20, 30, 40, and 50 mV·s^−1^, and (**d**) specific capacitance of PC, PZCC-1, and PZCC-2 at different densities of 5, 10, 20, 30, 40, and 50 mV·s^−1^.

**Figure 6:**
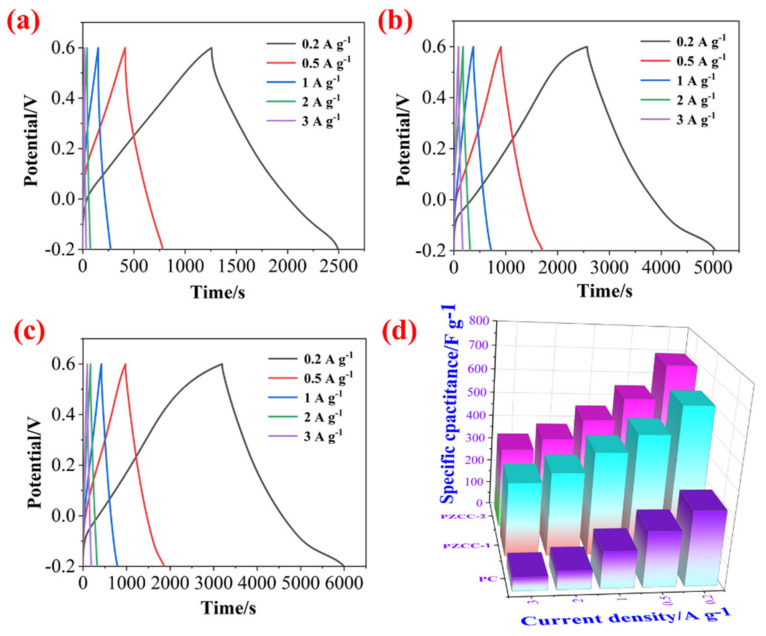
GCD profiles of PC (**a**), PZCC-1 (**b**), and PZCC-2 (**c**) electrodes at different current densities of 0.2, 0.5, 1, 2, and 3 A·g^−1^. (**d**) Comparative profiles of specific capacitance for PC, PZCC-1, and PZCC-2.

**Figure 7:**
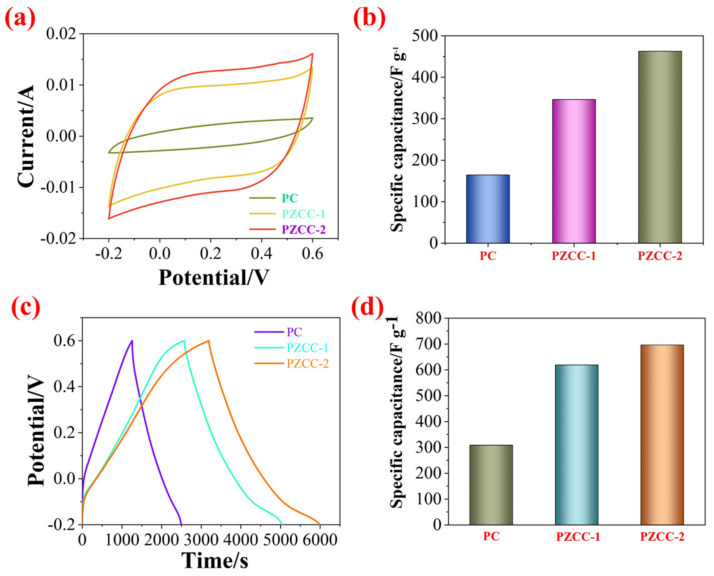
(**a**) Comparative CV profiles of PC, PZCC-1, and PZCC-2 at a scan rate of 5 mV·s^−1^, (**b**) specific capacitance of PC, PZCC-1, and PZCC-2 at a current density of 5 mV^−1^, (**c**) comparative GCD profiles of PC, PZCC-1, and PZCC-2 at a current density of 0.2 A·g^−1^, and (**d**) specific capacitance of PC, PZCC-1, and PZCC-2 at a current density of 0.2 A·g^−1^.

In the three-electrode system, the CV and GCD tests were carried out in 0.6 M H_2_SO_4_ electrolyte over the potential window of (−0.2)–0.6 V to evaluate the capacitance performance of hybrid electrode materials. As shown in Figure 5, the CV curves of PC, PZCC-1, and PZCC-2 present spindle shapes at higher scan rates, suggesting that the electrode material is not the only existence of electric double layer capacitance behavior. Moreover, the curves of PZCC-1 and PZCC-2 electrode materials were more rectangular than that of PC, indicating a lower charge transfer resistance, which is beneficial to obtain better electrochemical capacitance behavior. This opinion is also confirmed in EIS curves. The specific capacitance of electrode materials at different scan rates ranging from 5 to 50 mV·s^−1^ are obtained via integrating CV curves. The result shows that the specific capacitance of PC is 164.41, 117.64, 82.78, 68.53, 59.38, and 52.33 F·g^−1^. The specific capacitance of PZCC-1 is 346.45, 308.59, 247.01, 205.72, 174.77, and 95.92 F·g^−1^. The specific capacitance of PZCC-2 is 462.65, 356.99, 267.42, 211.04, 173.12, and 147.03 F·g^−1^. The PZCC-2 possesses a higher capacitance behavior than that of PC electrode material. This may be attributed to the more rapid charge transfer at the electrode/electrolyte interface, or the more efficient electrochemical behavior of the interface due to the change of the morphology and structure of PPy itself. As described above, GCD tests were also performed in order to study the capacitance performance of the electrode materials. As shown in Figure 6, the charging/discharging curves of all electrode materials show certain symmetry, indicating the reversibility of electrode materials. According to the various current densities of 0.2, 0.5, 1, 2, and 3 A·g^−1^, we calculated the capacitance of electrode materials. The specific capacitance of PC is 308.75, 228.31, 151.5, 76.75, and 55.87 F·g^−1^, the specific capacitance of PZCC-1 is 618.95, 500.56, 427.75, 345.00, and 304.88 F·g^−1^, and the specific capacitance of PZCC-2 is 696.65, 553.88, 461.88, 379.25, and 333.75 F·g^−1^, respectively. Besides, the comparative CV and GCD profiles of PC, PZCC-1, and PZCC-2 electrode materials at a scan rate of 5 mV^−1^ and a current density of 0.2 A·g^−1^ are shown in Figure 7, which can make it easier to see the significant differences and different reaction systems that also affect the PPy hybrid electrode materials. Compared to other CPs-based composites [47,48,49,50,51,52,53,54], the prepared electrode has better specific capacitance, which shows that this method is superior. The results indicate that the incorporation of ZIF-67 derived oxyhydroxide can improve the interface between CFs and PPy, which is beneficial to anchor the electrode material for more excellent electrochemical performance.

**Figure 8:**
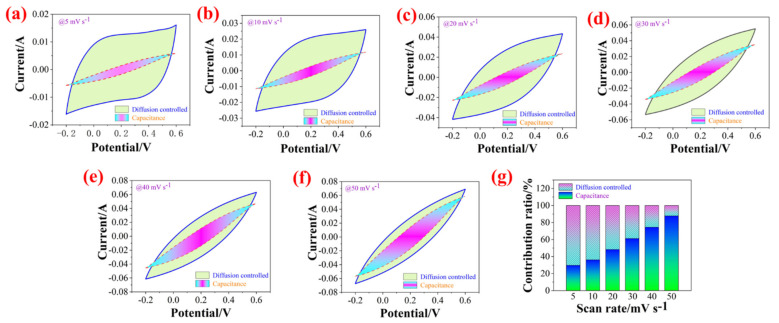
Diffusion controlled and capacitance CV profiles for PZCC-2 electrodes at different scan rates of 5, 10, 20, 30, 40, and 50 mV·s^−1^ (**a**–**f**), and (**g**) contribution ratio of diffusion controlled and capacitance for PZCC-2 electrode material at different scan rates of 5, 10, 20, 30, 40, and 50 mV·s^−1^.

To further explore the electrochemical reaction kinetics of electrode materials, in this paper, we took PZCC-2 as an example to analyze its CV curves. The total capacitance can be divided into surface capacitance and diffusion-controlled contribution at a certain scanning rate according to the following formula:

(6) $$i=k_{1}v+k_{2}v^{1/2}$$

where *k*_1_*ν* and *k*_2_*ν* are attributed to surface capacitive effect and diffusion-controlled process, respectively. Here, when *k*_2_ is zero, we obtain *k*_1_ value via calculating the current and scan rate ratio of CV curve at different scan rates. Then, the surface capacitance contributions of CV curves are integrated to obtain the contribution ratio of surface capacitance and diffusion-controlled capacitance at a different scan rate as shown in Figure 8. The surface capacitance contributions are 29.66%, 36.13%, 48.24%, 61.13%, 74.51%, and 87.74%, indicating that the capacitance is mainly reflected in the surface capacitance behavior at high scan rate, it is also difficult to reflect the real capacitance behavior of electrode material.

**Figure 9:**
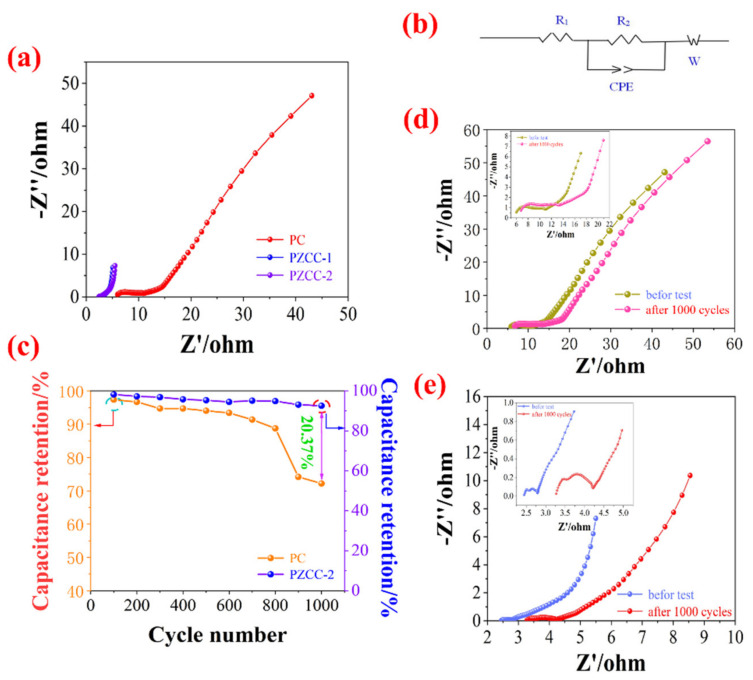
(**a**) Comparative Nyquist plots of PC, PZCC-1, and PZCC-2 electrode materials, (**b**) EIS circuit of electrode materials, (**c**) capacitance retention of PC and PZCC-2 electrode materials, and (**d**,**e**) Nyquist plots of PC and PZCC-2 electrode materials before and after 1000 cycles.

The EIS measurements demonstrate the far better conductivity of PZCC-1 and PZCC-2 than that of PC electrode materials, as shown in Figure 9a, and we also simulated the EIS plots based on it. The result is shown in Figure 9b, demonstrating that the electrochemical process of electrode materials includes three parts of equivalent series resistance (ESR), charge transfer resistance (Rct), and Warburg (W) diffusion. From EIS plots, the resistance value of PC is about 5.9 Ω, which is higher than those of PZCC-1 (~2.8 Ω), PZCC-2 (~2.5 Ω) and the previous study (~3.5 Ω) [36], indicating faster electron transfer of PZCC-2 electrode material at the interface between electrode and electrolyte. The straight line at the low frequency of PZCC-2 is closer to the Y-axis, suggesting better ion diffusion from the electrolyte to the electrode. All the results support the better specific capacitance performance of PZCC-2 than that of PC electrode material.

The service life is very important in the evaluation of the electrochemical performance of the electrode materials. The electric double layer electrode material has an excellent cyclic stability due to its own energy storage characteristics. For the CPs@CFs pseudo-capacitance electrode material, the continuous expansion or collapse of the conductive polymer will result in the poor cycle stability of the composite material during the electrochemical cycle, which is not conducive to its further processing and utilization. Here, we use ZIF-67 as the precursor to prepare ZIF-67-derived cobalt oxyhydroxide@CFs composite materials by further oxidation, and then load the PC conductive polymer on the composite material via the in-situ method. The result is shown in Figure 9c, the capacitance retention of PZCC-2 hybrid electrode material (92.56%) is higher than that of PC electrode materials (72.19%), suggesting that the introduction of ZIF-67-derived cobalt oxyhydroxide improved the intrinsic resistance and interface charge transfer. The EIS plots of PC and PZCC-2 electrodes before and after 1000 cycles are shown in Figure 9d,e. The equivalent series resistances of PC and PZCC-2 electrodes after 1000 cycles are about 3.9 Ω and 6.8 Ω, which are higher than those of PC and PZCC-2 electrodes before cycle. Similarly, the electrodes have higher charge transfer resistance and longer Weber liner, which also explains the decline of capacitance retention for PC and PZCC-2 electrodes.

**Figure 10:**
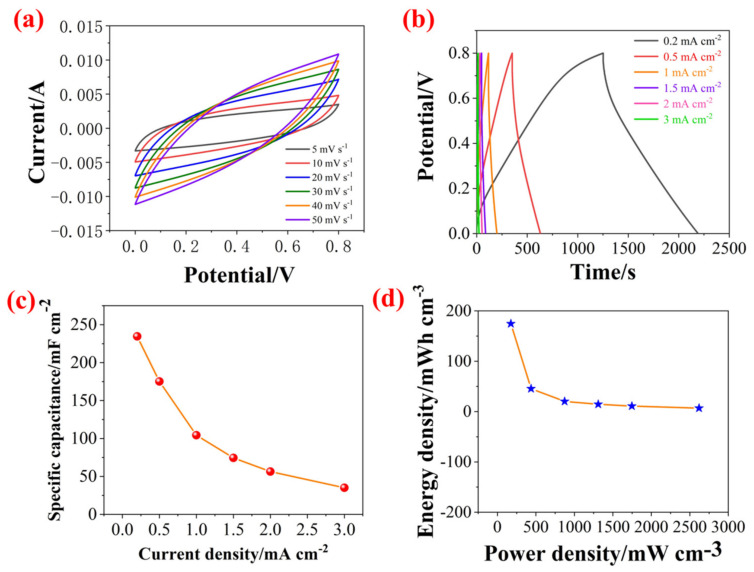
(**a**) CV curves of supercapacitors (SCs) based on PZCC-2 electrode materials at various scan rates from 5 to 50 mV s^−1^, (**b**) GCD curves SCs based on PZCC-2 electrode materials at different current densities from 0.2 to 3 mA cm^−2^, (**c**) specific capacitance of SCs at different current densities, and (**d**) energy density–power density curves of SCs.

To investigate the electrochemical performance of PZCC-2-SCs, the CV and GCD were performed at various scan rates and current densities over the potential window of 0–0.8 V, and the corresponding results are shown in Figure 10. The specific capacitance is 234.50, 175.19, 104.25, 74.49, 56.30, and 35.03 mF·cm^−2^. Then, the volumetric energy density (E) and power density were calculated according to the equations of (4) and (5). The PZCC-2-SCs can provide an energy density of 45.51 mWh cm^−3^ at a power density of 174.67 mW cm^−3^, which is higher than previous report [53,54], suggesting the potential application in energy storage area.

In conclusion, the PPy@ZIF-67 derived cobalt oxyhydroxide@CFs hybrid electrode material can be applied in energy storage field for the following reasons. Firstly, CFs as flexible substrate materials are the most abundant resource on earth, and have excellent characteristics, such as flexibility, porous structures, and wettability, etc. Secondly, the PPy conductive polymer possesses superior conductivity and redox pseudo-capacitance features. Thirdly, the introduction of ZIF-67-derived cobalt oxyhydroxide is beneficial to anchor PPy via the chemical bond force of Co–Nx, which can improve the intrinsic resistance and interface charge transfer, which makes PPy@CFs composites have better specific capacitance and cyclic stability.

## 4. Conclusions

In summary, we designed a feasible method to prepare ZIF-67-derived cobalt oxyhydroxide with high crystallinity and nanoscale for enhancing PPy@CFs composite. The incorporation of ZIF-67-derived cobalt oxyhydroxide in composite improves the hyrdrophilicity and conductivity (resistance value of ~2.5 Ω) of hybrid electrode, and hence obtains higher specific capacitance (696.65 F·g^−1^) and capacitance retention (92.56%). Besides, The PZCC-SC can provide an energy density of 45.51 mWh·cm^−3^ at a power density of 174.67 mW·cm^−3^, which is higher than that of previous report. This work demonstrated that the modification of PPy@CFs electrode with ZIF-67-derived cobalt oxyhydroxide could be an effective approach to fabricate CPs@CFs electrodes with excellent performance, which will facilitate their application in energy storage area.

## Data Availability

The authors confirm that the data supporting the findings of this study are available within the article.
